# Constitutively active androgen receptor splice variants *AR-V3*, *AR-V7* and *AR-V9* are co-expressed in castration-resistant prostate cancer metastases

**DOI:** 10.1038/s41416-018-0172-0

**Published:** 2018-07-10

**Authors:** Heini M. L. Kallio, Reija Hieta, Leena Latonen, Anniina Brofeldt, Matti Annala, Kati Kivinummi, Teuvo L. Tammela, Matti Nykter, William B. Isaacs, Hans G. Lilja, G. Steven Bova, Tapio Visakorpi

**Affiliations:** 1 Prostate Cancer Research Center, Faculty of Medicine and Life Sciences and BioMediTech Institute, University of Tampere, Tampere, Finland; 2 Department of Urology, University of Tampere, Tampere University Hospital, Tampere, Finland; 3 The James Buchanan Brady Urological Institute, Johns Hopkins University School of Medicine, Baltimore, MD USA; 4 Departments of Surgery (Urology), Laboratory Medicine and Medicine, Memorial Sloan-Kettering Cancer Center, New York, NY USA; 5 Nuffield Department of Surgical Sciences, University of Oxford, Oxford, UK; 6 Department of Translational Medicine, Lund University, Malmö, Sweden; 7 Prostate Cancer Research Center, Faculty of Medicine and Life Sciences and BioMediTech Institute, University of Tampere, Tampere, Finland; 8 Fimlab Laboratories, Tampere University Hospital, Tampere, Finland

**Keywords:** Prostate cancer, Next-generation sequencing, Data processing

## Abstract

**Background:**

A significant subset of prostate cancer (PC) patients with a castration-resistant form of the disease (CRPC) show primary resistance to androgen receptor (AR)-targeting drugs developed against CRPC. As one explanation could be the expression of constitutively active androgen receptor splice variants (AR-Vs), our current objectives were to study *AR-V*s and other *AR* aberrations to better understand the emergence of CRPC.

**Methods:**

We analysed specimens from different stages of prostate cancer by next-generation sequencing and immunohistochemistry.

**Results:**

*AR* mutations and copy number variations were detected only in CRPC specimens. Genomic structural rearrangements of *AR* were observed in 5/30 metastatic CRPC patients, but they were not associated with expression of previously known *AR-V*s. The predominant *AR-V*s detected were *AR-V3*, *AR-V7* and *AR-V9*, with the expression levels being significantly higher in CRPC cases compared to prostatectomy samples. Out of 25 CRPC metastases that expressed any *AR* variant, 17 cases harboured expression of all three of these *AR-V*s. AR-V7 protein expression was highly heterogeneous and higher in CRPC compared to hormone-naïve tumours.

**Conclusions:**

*AR-V3*, *AR-V7* and *AR-V9* are co-expressed in CRPC metastases highlighting the fact that inhibiting AR function via regions common to all AR-Vs is likely to provide additional benefit to patients with CRPC.

## Introduction

Prostate cancer (PC) is the most common malignancy and third most common cause of cancer-related death among men in Europe. Androgens are required for the normal development of prostate tissue and exert their effects through androgen receptor (AR)-mediated signalling, but also have important role during PC emergence and progression. Most PCs grow slowly and are curable by surgery and radiation when confined to the prostate. In contrast, treatment of PCs that have spread outside the prostate usually includes manipulation of the AR signalling axis; androgen-deprivation therapy (ADT) either by surgical or chemical castration. However, during currently available ADT, lethal castration-resistant form of PC (CRPC) will eventually emerge after a variable period of time. Even though the exact mechanism by which CRPC develops remains to be fully understood, several mechanisms of castration resistance have been identified such as *AR* gene amplification,^1,2^ point mutations in *AR* gene^3,4^ and induction of steroidogenesis in CRPC cells.^5–7^ *AR* gene amplification has been demonstrated in approximately 30% of CRPCs.^1^ Cancers with *AR* amplification have been shown to respond better to second-line maximal androgen blockade compared to tumours without the amplification, however, the response was short-lived.^8^ Also *AR* mutations are rare even at CRPC stage being present in approximately 10–30% of cases.^4^ These mutations are almost always associated with diverse gains-of-function and about 45% of the mutations occur in the ligand-binding domain.^9^ *AR* mutations can broaden ligand specificity to alternative steroid hormones, hypersensitise the receptor to castrate levels of androgens or lead to resistance to current forms of treatment making AR active even in the presence of anti-androgens.^10^

Importantly, androgen signalling remains active even in the CRPC stage.^11,12^ The established concept of sustained AR signalling during CRPC has led to the clinical development of second-generation AR-targeting drugs enzalutamide and abiraterone that target the ligand-binding domain of AR directly and indirectly, respectively. Enzalutamide is an AR antagonist, whereas abiraterone is a CYP17 inhibitor approved by the US Food and Drug Administration (FDA) for the treatment of metastatic CRPC. Several studies have shown that presence of *AR* amplification or *AR* mutations in plasma samples is associated with worse outcome with enzalutamide and abiraterone.^13–17^ Furthermore, a significant subset of patients show primary resistance to these agents with respect to PSA (prostate-specific antigen) level,^18^ and among patients who initially respond, nearly all eventually develop acquired resistance.

One potential explanation for the resistance to first-generation and second-generation AR-targeted therapies is the presence of AR splice variants (AR-Vs). AR-Vs are alternatively spliced isoforms of the *AR* mRNA usually resulting in truncated AR protein product. The key domains shared among wild-type full-length AR (AR-FL) and all AR-Vs are the NH_2_-terminal transactivating domain (NTD) and DNA-binding domain (DBD). However, AR-Vs lack variable portions of the COOH-terminal domain including the ligand-binding domain (LBD).^19–21^ In spite of the fact that AR-Vs are unable to bind a ligand, they are constitutively active as transcription factors and capable of activating target genes.^22^

To date, at least 22 AR-Vs have been discovered in CRPC specimens.^23^ AR-V7 is the most clinically relevant variant as it is most frequently observed and the most abundant AR-V in clinical specimens. In addition, AR-V7 is the only variant that can be detected reproducibly at both the mRNA and protein levels. Moreover, detection of *AR-V7* mRNA in circulating tumour cells (CTCs) and peripheral whole blood from CRPC patients treated with enzalutamide or abiraterone has been implicated in primary resistance and shorter progression-free and overall survival.^24–27^ Interestingly, the prevalence of *AR-V7* was shown to be higher in enzalutamide-treated men who had previously received abiraterone and in abiraterone-treated men who had previously received enzalutamide.^25^ These findings were supported by an independent study that also utilised CTC-based RT-PCR assay.^28^ In this prospective study, it was shown that PSA response rate to abiraterone or enzalutamide was 7% among *AR-V7-*positive patients and 63% among *AR-V7*-negative patients. Another recent study demonstrated that *AR-V7* detection in plasma-derived exosomal RNA strongly predicts resistance to enzalutamide or abiraterone in CRPC patients.^29^ Although these studies implicate that AR-V7 could be used as a treatment-specific biomarker, it is likely that other AR-Vs also play a role in the development of CRPC. For example, it was recently reported that *AR-V9* is often co-expressed with *AR-V7* in CRPC metastases, and predicts primary resistance to abiraterone.^30^

Recently, genomic structural rearrangements of *AR* (*AR*-GSRs) were established as a new class of *AR* gene alteration occurring in one third of CRPC-stage specimens.^31^ This work showed that the presence of *AR*-GSRs at high variant allele frequency was associated with outlier, tumour-specific expression of rearrangement-dependent *AR-V* species that displayed androgen-independent and enzalutamide-resistant transcriptional activity. However, contrary to the prior studies in cell lines,^32,33^ *AR*-GSRs were not associated with the *AR-V7* expression levels in metastatic CRPC tissue.^31^ Another recent study utilising peripheral blood collected from patients with CRPC detected intra-*AR* structural variation in 15/30 patients of whom 14 expressed *AR-V*s.^34^ Of note, most of the *AR-V* -positive patients expressed multiple *AR-V*s, with *AR-V7* being the most frequently occurring splice variant. However, *AR-V3* was the most abundantly expressed *AR* splice variant. According to this study the presence of any *AR-V* was associated with shorter progression-free survival after second-line endocrine treatment compared to patients that did not harbour *AR-V*s.^34^ Furthermore, in another recent investigation *AR*-GSRs in circulating tumour DNA were shown to associate with primary resistance to enzalutamide or abiraterone also in treatment-naïve CRPC patients with metastatic disease.^13^

Our aim was to study *AR* splice variants, rearrangements, mutations and copy-number variations (CNVs) in different stages of PC to better understand the emergence of CRPC. We used multiple sample cohorts representing hormone-naïve PCs and lymph node metastases as well as locally recurrent and metastatic CRPCs. We first employed whole-genome and whole-transcriptome sequencing followed by targeted *AR* sequencing panels allowing deeper sequence coverage. In particular, our aim was to confirm whether *AR-V*s are expressed in higher levels in CRPC samples compared to earlier stage cancers. In addition, we wished to elucidate to what extent *AR-V* expression is due to the aberrant splicing and, on the other hand, *AR* gene rearrangements. We also wanted to study the association between *AR-V* and *AR-FL* expression; and to find out whether *AR-V* expression affects the expression of AR-regulated genes.

## Materials and methods

### Sample sets

Two different sample sets utilised in the study are shown in Table 1 and are described in more detail in Supplementary Table S1. The sample set 1 contained freshly frozen tissue specimens from benign prostatic hyperplasia (BPH) (*n* = 12), hormone-naïve PC (*n* = 30) and locally recurrent CRPC (*n* = 13) with clinicopathological characteristics of PC cases and prior treatments of CRPC cases being shown in Supplementary Table S1. BPH samples were obtained by radical prostatectomy, cystoprostatectomy and by transurethral resection of the prostate. Hormone-naïve PC samples were obtained by radical prostatectomy and locally recurrent CRPCs by transurethral resection of the prostate. Histological evaluation and Gleason grading were performed by a pathologist based on haematoxylin/eosin stained slides. All samples contained a minimum of 70% cancerous or hyperplastic cells. DNA and RNA were isolated simultaneously using an AllPrep DNA/RNA Mini Kit (Qiagen, Valencia, CA, USA), according to manufacturer’s protocol. For certain samples, additional total RNA was isolated using Trizol (Invitrogen, Carlsbad, CA, USA) extraction according to manufacturer’s protocol. Three CRPC samples had RNA extracted using both Trizol and Qiagen AllPrep. Integrity was checked using Bioanalyzer (Agilent Technologies, Santa Clara, CA, USA).

**Table 1:** Sample sets utilised in the study

	Number of samples	Origin	Subgroups	Sequencing method
Sample set 1	55	Prostatectomy specimens of non-treated cancer, transurethral resection specimens of locally recurrent CRPC and BPH specimens obtained by several methods	BPH (12), PC (30), locally recurrent CRPC (13)	Whole genome and whole transcriptome/HiSeq
Sample set 2	80	Prostatectomy specimens of non-treated cancer, lymph node metastases collected during lymphadenectomy, metastatic CRPC specimens and noncancerous control specimens collected from 30 individuals at autopsy	PC (24), lymph node metastasis (8), metastatic CRPC (30), noncancerous control (18)	SureSelect targeted DNA and targeted RNA/MiSeq

*BPH* benign prostatic hyperplasia, *PC* prostate cancer, *CRPC* castration-resistant prostate cancer

The sample set 2 consisted of 24 additional hormone-naïve PCs removed by prostatectomy, of which six specimens were also included in sample set 1, eight lymph node metastases obtained at lymphadenectomy and 30 metastatic CRPC specimens obtained at autopsy (clinicopathological characteristics of the cases are shown in Supplementary Table S1). Hormone-naïve PC samples contained a minimum of 60% cancerous or hyperplastic cells and were processed as described in the previous section.

Portions of the metastatic cancer tissue from pelvic lymphatic metastasis obtained at lymphadenectomy were used for this study. None of the eight patients had undergone ADT, chemotherapy or radiation therapy prior to this surgery. Precise histological control was achieved for all tissues studied in this group using the following protocol. Serial cryostat sectioning was used to identify portions of the sample containing a lower fraction of tumour cells. These areas were manually microdissected from the tissue block every 300 µm based on H&E stained slide visual analysis. The tumour cell fraction was 70% or greater by histologic visual estimation. DNA purification was performed as described previously.^35^ Total RNA was isolated using an AllPrep DNA/RNA/miRNA Universal Kit (Qiagen, Valencia, CA, USA) according to manufacturer’s instructions. The integrity of isolated RNA was confirmed using Fragment Analyzer (Advanced Analytical Technologies, Ankeny, IA, USA).

Metastatic CRPC specimens were obtained from 30 men who participated in the PELICAN (Project to ELIminate lethal CANcer) integrated clinical-molecular autopsy study of metastatic PC (a detailed sample list is shown in Supplementary Table S1). Androgen axis and corticosteroid clinical treatments are listed in the Supplementary Table S1. All metastases (one metastasis per patient) and noncancerous (normal, NL) control samples studied were obtained at autopsy. Isolated frozen tissue samples were serial cryostat microdissected for histological tumour purity >75%, and high-molecular-weight DNA was isolated using proteinase K digestion and phenol/chloroform extraction. Total RNA was isolated using an AllPrep DNA/RNA/miRNA Universal Kit (Qiagen, Valencia, CA, USA) according to manufacturer’s instructions. The integrity of isolated RNA was confirmed using Fragment Analyzer (Advanced Analytical Technologies, Ankeny, IA, USA).

Low-coverage (4–6×) whole-genome DNA sequencing and whole-transcriptome sequencing (applied to sample set 1) have been described before.^36^

### Targeted *AR* DNA assay library construction and sequencing (applied to sample set 2)

A custom DNA sequencing panel was designed to cover all *AR* exons and introns. In addition, *FOXA1*  exons  and *SPOP* exons 6-7 were included in the panel. Targeted sequence enrichment was performed using the SureSelect^XT^ Target Enrichment System (Agilent Technologies, Santa Clara, CA, USA) according to manufacturer’s instructions. Briefly, 200 ng of genomic DNA was fragmented using Covaris® (Covaris, MA, USA) to yield a fragment size of 150–200 bp. End repair, addition of the 3’-dA overhang, ligation of indexing-specific adaptors, hybridisation to custom RNA baits, hybrid capture selection and index tagging were performed according to the Illumina paired-end sequencing library protocol. All recommended quality controls were performed between steps. The multiplexed samples were sequenced on the Illumina Miseq platform using 150 bp paired-end reads.

### Targeted *AR* RNA assay library construction and sequencing (applied to sample set 2)

*AR* and five androgen-responsive genes (*KLK3*, *FKBP5*, *TMPRSS2*, *ACPP* and *SLC45A3*) were targeted for capture and sequencing. In addition, three house-keeping genes *TBP*, *STARD7* and *DDX1* were included for normalisation purposes. This custom RNA sequencing panel was designed to cover all *AR* exons and nonrepetitive intronic regions to enable investigation of most common *AR* splicing variants (*AR-V3*, *AR-V4*, *AR-V5*, *AR-V6*, *AR-V7*, *AR-V9*, *AR-V12* and *AR-45*); other genes were covered less intensively (one or five amplicons per gene). Targeted sequence enrichment was performed using the SureSelect^XT^ RNA Target Enrichment System (Agilent Technologies, Santa Clara, CA, USA) according to manufacturer’s instructions. Briefly, poly(A) RNA was purified from 1 µg of total RNA and fragmented chemically. In the following steps, samples were prepared using SureSelect Strand-Specific RNA Library Prep Kit to obtain adaptor-ligated cDNA library amplicons. Finally, hybridisation to custom RNA baits, hybrid capture selection and index tagging were performed. All the AMPure XP bead purification steps were conducted as instructed. The multiplexed samples were sequenced on the Illumina Miseq platform using 150 bp paired-end reads. The following modifications were made to the protocol if RNA was highly degraded (RQN < 6 determined by Fragment Analyzer) as recommended by Agilent Technologies: (1) Instead of poly(A) RNA purification from 1 µg total RNA, Ribo-Zero Gold Magnetic Kit (Illumina, San Diego, CA, USA) was used to remove rRNA from 2 µg of total RNA. (2) Instead of fragmenting the purified RNA at 94 °C for 8 min, RNA was denatured at 65 °C for 5 min. (3) All AMPure XP bead purification steps were performed using 1.8:1 bead volume to sample volume ratio. (4) Instead of 13 cycles in the pre-capture PCR, the number of cycles was increased to 14.

### Validation of the targeted sequencing panels

Targeted custom SureSelect sequencing panels were validated by evaluating their performance in detecting *AR* aberrations in comparison to our previously published whole-genome DNA-seq data^37,38^ and whole-transcriptome RNA-seq data from this study. There was a good concordance in mutation detection between SureSelect DNA panel and previously analysed data from 22Rv1 cell line sample and metastatic CRPC samples from patients A21, A22 and A24. SureSelect DNA-seq detected the previously found H875Y mutation from 22Rv1 cell line, L702H mutation from liver metastasis from patient A21 as well as T878A mutation from a pelvic lymph node metastasis from patient A22, and from a right rib metastasis from patient A24.^37,38^ The data from AR splicing variant analysis also showed good accordance between SureSelect RNA panel and whole-transcriptome RNA-seq in three PC cell lines and two patient samples (Supplementary Fig. S1). It should be noted that SureSelect RNA assay was more sensitive in detecting *AR-V9* than whole-transcriptome RNA-seq.

### Bioinformatics

For analysis of targeted DNA-seq data, Illumina MiSeq reads were aligned to GRCh37 (hg19) genome using Bowtie2.^39^ *AR*, *FOXA1* and *SPOP* variants were called using an in-house pipeline that utilises samtools mpileup.^40^ Filtered variants were annotated using the ANNOVAR software.^41^ Variants in dataset 1 were analysed from the whole-transcriptome sequencing data similarly.

*AR* copy numbers were analysed by calculating aligned read counts within overlapping 400 bp windows along the targeted regions using bedtools.^42^ The median of all AR bait coverage ratios that were obtained by dividing each normalised bait coverage value by the median of all values was used as the estimate of *AR* copy number. Chromosomal rearrangements were called using the in-house Breakfast algorithm that looks for paired-end reads and individual mates overlapping a chromosomal breakpoint.

For *AR* splice variant analysis using targeted or WTS RNA-seq data, Illumina MiSeq reads or HiSeq reads were aligned to an indexed reference fasta file containing unique signature sequences for various *AR-V*s and *AR-FL*. The signatures consisted of 130 bp of the 3’ end of upstream exon and 130 bp of the 5’ end of downstream exon of a given unique splice junction (Supplementary Table S2). Relative *AR-V* expression was estimated as the percentage of all *AR* transcripts by dividing the number of reads aligned to a given *AR-V* signature by the total number of reads aligning to all the splice junctions containing the same upstream exon.

Expression levels of known AR-regulated genes were determined by aligning RNA-seq reads to GRCh37 genome using TopHat2.^43^ *Z*-scores were calculated from the normalised read counts, and AR-signalling score was computed as the sum of the *Z*-scores of all AR-regulated genes. Full bioinformatics methods are described in the Supplementary methods.

### Immunohistochemistry

Formalin-fixed, paraffin-embedded tumour microarrays of hormone-naïve PC, locally recurrent CRPC and metastatic CRPC (described in Leinonen et al. 2013) were used. Immunohistochemistry for AR (with N-terminal antibody recognising full-length AR and the variants) has been previously described.^44^ For AR-V7-specific staining, sections were deparaffinised, and antigen retrieval was performed by using Tris-EDTA buffer 0.05% Tween-20 (pH 9) at +98 °C for 15 min. The staining was performed by Lab Vision Autostainer (ThermoFischer Scientific Inc., Waltham, MA, USA). The primary antibody Anti-Androgen Receptor (AR-V7 specific) Rabbit Monoclonal Antibody [RM7] (RevMAb Biosciences, San Francisco, CA, USA) and secondary antibody (N-Histofine® Simple Stain MAX PO; Nichirei, Tokyo, Japan) were used. ImmPACT DAB (Vector Laboratories, Burlingame, CA, USA) was used as a chromogen. The sections were counterstained with haematoxylin and mounted with DPX mounting medium (Sigma-Aldrich). The percentage of AR-V7-positive cells between PC and CRPC groups was statistically assessed with Mann-Whitney test.

## Results

### *AR* mutations and CNVs are detected only in CRPC cases

**Fig. 1:**
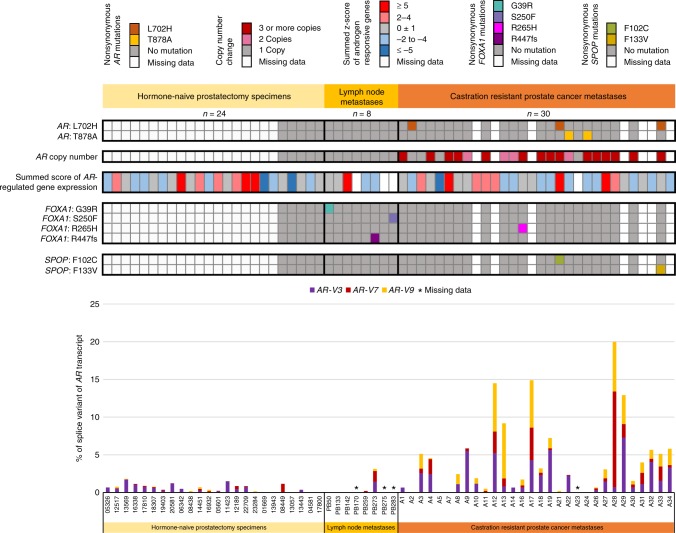
Combined DNA and RNA sequencing data from sample set 2 assayed by targeted SureSelect *AR* sequencing. *AR* mutations, copy-number alterations, summed score of AR-regulated gene expression and *AR-V* expression level as a fraction of *AR* transcript are shown. *AR-V* fractions are shown as CI95 lower bound values. Additionally, *FOXA1* and *SPOP* mutation status is included

Since the mechanisms leading to emergence of CRPC are still largely unknown, we wanted to study the expression of *AR* splicing variants and other *AR* aberrations in tandem during different stages of PC. For this purpose, we performed low-coverage whole-genome DNA sequencing and whole-transcriptome sequencing in sample set 1 that included BPH specimens, hormone-naïve PC from prostatectomies and locally recurrent CRPCs (Supplementary Fig. S2). In addition, we performed targeted *AR* DNA and RNA sequencing in sample set 2 that contained hormone-naïve PC from prostatectomies and lymph node metastases as well as CRPC metastases (Fig. 1).

First, we wanted to analyse the status of *AR* mutations and CNVs across widely diverse set of samples to better understand their potential link to *AR-V* expression. As expected, *AR* mutations and CNVs were detected only in locally recurrent and metastatic CRPC specimens (Fig. 1 and Supplementary Fig. S2, upper panels). T878A mutation that has been shown to confer agonist activity of flutamide on the AR^45–47^ was found in 1/13 (8%) of locally recurrent CRPC specimens and in 2/23 (9%) of metastatic CRPC specimens. L702H mutation that converts glucocorticoids to AR agonists^48,49^ was found in 3/23 (13%) of metastatic CRPC specimens. Indeed, all three patients harbouring L702H mutation had been treated with glucocorticoids (Supplementary Table S1; a detailed treatment history is shown for patients having *AR* mutations). Copy-number gains (>1 copy of *AR*) or amplifications (>2 *AR* copies) were observed in 4/9 (44%) of locally recurrent CRPC specimens and in 19/23 (83%) of metastatic CRPC specimens, respectively (Fig. 1 and Supplementary Fig. S2). It should be noted that there were striking differences in *AR* copy numbers in metastatic CRPC specimens; for example, the lesion from patient A7 had six *AR* copies, whereas the lesion from patient A4 had as many as 68 *AR* copies. In four metastatic CRPC specimens, *AR* gain or amplification co-occurred with *AR* mutation. Since a large body of data, including our current investigation, has established that there are no mutations or copy number aberrations of *AR* in untreated PCs, majority of prostatectomy specimens in sample set 2 were not assayed with the targeted SureSelect DNA panel (Fig. 1). Additionally, data from targeted DNA assay are missing from those metastatic CRPC specimens of which DNA was not available (Fig. 1). The overall average coverage of the targeted regions in the samples ranged from 109× to 1829×, with the average coverage in the *AR* region being somewhat higher (114×–3358×).

### The expression of *AR-V*s is highest in CRPCs and associates with expression of *AR-FL*

Next we studied the presence of known *AR-V*s that were detected from the RNA-seq data by aligning the reads against indexed *AR-V* signature sequence file containing exon-exon junction sequences unique to every *AR-V* under investigation (an example of RNA-seq read alignment of patient A17 is visualised in Supplementary Fig. S3). The *AR-V*s detected by our assays included *AR-V3*, *AR-V4*, *AR-V5*, *AR-V6*, *AR-V7* and *AR-V9*. The expression levels of *AR-V4*, *AR-V5* and *AR-V6* were negligible in comparison to *AR-V3*, *AR-V7* and *AR-V9*, and were mainly observed in CRPC metastases. In sample set 1 run by whole-transcriptome RNA-seq, BPH specimens were mainly devoid of *AR-V* expression. Instead, *AR-V3* and *AR-V7* expression were detected in both hormone-naïve PC from prostatectomies and locally recurrent CRPCs with minimal co-expression of *AR-V9* (Supplementary Fig. S2). Whereas the expression of *AR-V3* was quite similar in the two different categories of samples, higher *AR-V7* expression levels were detected in locally recurrent CRPCs as compared with hormone-naïve PC from prostatectomies. Since the depth of the whole-transcriptome sequencing was not satisfactory (average per-base sequence coverage ranged from 14×–137×) in terms of reliable detection of *AR* variants, we also performed targeted RNA sequencing of the *AR*, which provided an average coverage range from 95× to 2247× utilising sample set 2 (Fig. 1). In sample set 2, not only the expression level of *AR-V7* but also the expression levels of *AR-V3* and *AR-V9* were higher in metastatic lesions from CRPC cases compared to hormone-naïve PC from prostatectomy (Fig. 1). The differences were statistically significant for either variant alone (Supplementary Table S3) or when their expression fractions were combined (*p* = 0.0006, unpaired Wilcoxon rank sum test, Table 2). In addition, metastatic CRPC cases expressed significantly more *AR-V3*, *AR-V7* and *AR-V9* compared to non-androgen-deprived pelvic lymph node metastases (*p* = 0.0282, unpaired Wilcoxon rank sum test, Table 2). We also studied whether the expression of *AR-V*s is associated with the CNV status (neutral vs. duplicated/amplified *AR*) in sample set 2. There was a modest correlation when CNV status was compared to the combined expression levels of *AR-V3*, *AR-V7* and *AR-V9* (rho = 0.39, *p* = 0.005, Spearman’s rank correlation).

**Table 2:** Statistical comparison of combined expression of *AR-V3*, *AR-V7* and *AR-V9* in different sample types using two-tailed, unpaired Mann-Whitney *U* test

	Prostatectomy	Lymph node metastases
Prostatectomy	-	
Lymph node metastases	0.1765	-
CRPC metastases	0.0006***	0.0282*

*CRPC* castration-resistant prostate cancer.

^*^p < 0.05, ^***^p < 0.001. Statistically significant *p*-values are shown in bold

**Fig. 2:**
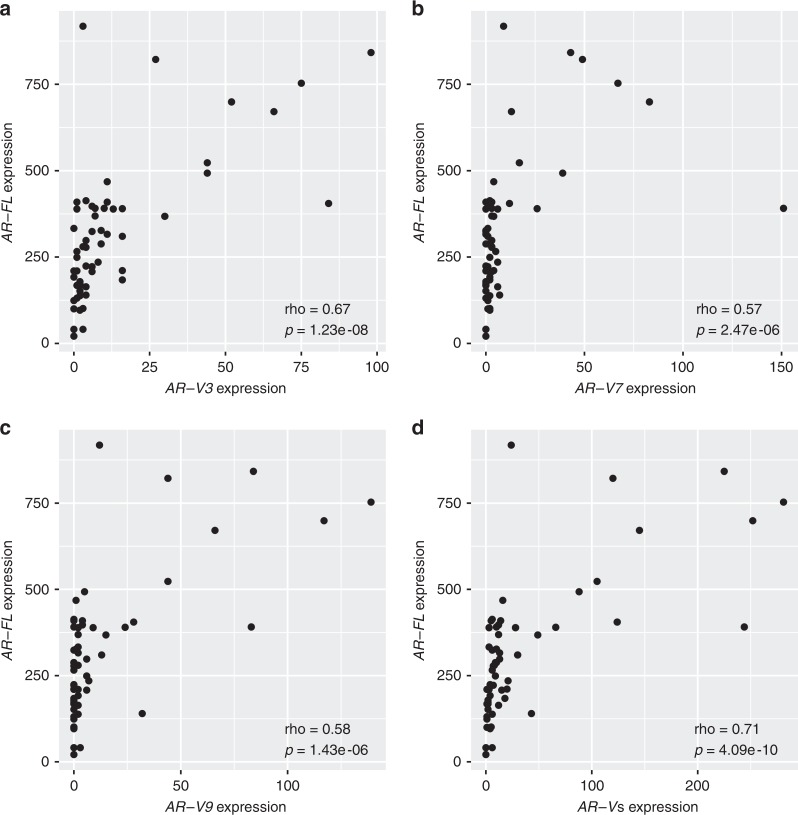
The correlation between *AR-FL* mRNA expression and mRNA expression of (**a**) *AR-V3*, (**b**) *AR-V7*, (**c**) *AR-V9*, (**d**) all three *AR-V*s combined utilising specimens from sample set 2. The counts of splice junction reads indicative of *AR-FL* or *AR-V*s are plotted in the *y*-axis and *x*-axis, respectively. Spearman’s rank correlation coefficients and *p* values computed via the asymptotic *t* approximation are also shown in the figures

**Fig. 3:**
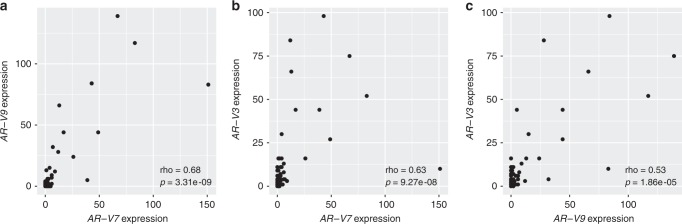
The correlation between (**a**) *AR-V7* and *AR-V9* mRNA expression, (**b**) *AR-V7* and *AR-V3* mRNA expression and (**c**) *AR-V9* and *AR-V3* mRNA expression utilising specimens from sample set 2. The counts of splice junction reads indicative of given *AR-V*s are plotted in the *y*-axis and *x*-axis. Spearman’s rank correlation coefficients and *p* values computed via the asymptotic *t* approximation are also shown in the figures

In sample set 2, *AR-FL* expression was threefold higher in metastatic CRPC compared to hormone-naïve PC from prostatectomy specimens, whereas in sample set 1, *AR-FL* was expressed fivefold higher in CRPC lesions than in prostatectomy samples. Notably, the expression of *AR-V3*, *AR-V7* and *AR-V9* was strongly associated with the levels of full-length *AR* in sample set 2 (Fig. 2) and in sample set 1 (Supplementary Fig. S4), suggesting that the expression of *AR* locus drives the expression of *AR-V*s both in hormone-naïve PC and in CRPC. Furthermore, there was strong and highly significant correlation between the expression of each individual *AR-V* compared to other *AR-V*s in sample set 2 (Fig. 3).

We also asked whether *AR* variant expression affects the expression of AR-regulated genes. This was done by calculating the summed *z*-score of five androgen-responsive genes (*KLK3*, *FKBP5*, *TMPRSS2*, *ACPP* and *SLC45A3*) (Fig. 1, Supplementary Fig. S2). *AR-V* expression was not associated with AR-regulated gene expression in sample set 2 when the proportion of fractions, when compared to *AR-FL*, of each *AR-V* or all *AR-V*s combined, were plotted against AR signalling score (Supplementary Fig. S5). In addition, we wanted to test whether *AR-V3*, *AR-V7* or *AR-V9* expression correlates in particular to *KLK3* expression. No correlation between either variant and *KLK3* was detected in metastatic CRPC specimens (Supplementary Fig. S6). We next studied mutation status of two AR-regulating genes, *FOXA1* and *SPOP*, in sample set 2. *FOXA1* mutations were found in 3/8 (38%) lymph node metastases and in 1/23 (4%) CRPC metastases, whereas *SPOP* mutations were detected in 1/8 (13%) lymph node metastases and in 2/23 (9%) CRPC metastases (Fig. 1). We did not find any association between *FOXA1* or *SPOP* mutation status and AR*-*regulated gene expression. All mutations found in this study and their variant allele frequencies are shown in Supplementary Table S4.

### *AR* genomic structural rearrangements occur in the context of amplified *AR*

*AR* genomic structural rearrangements (*AR*-GSRs) were recently identified as a novel class of *AR* alteration using both autopsy CRPC specimens and peripheral blood collected from CRPC patients.^31,34^ More importantly, the presence of *AR*-GSRs was associated with expression of *AR-V*s in both studies. To this end, we analysed *AR* DNA-seq data with our structural variant detection pipeline to identify *AR*-GSRs, defined as events having at least one breakpoint detected within the *AR* gene region. Average per-base sequence coverage of the *AR* gene region ranged from 114×–3358× and on average 78% of *AR* was covered by at least 10 reads (range 76–82%). We detected putative *AR*-GSRs in 5/30 metastatic CRPC patients who all harboured a highly amplified *AR* (Supplementary Table S5). All other sample types were negative for *AR*-GSRs when cut-off of 10 supporting split reads was used. It should be noted that none of the *AR*-GSRs occurred along with *AR* missense mutations. The break fusion junctions of *AR*-GSRs were variable, demonstrating several types of rearrangements including duplication, deletion, inversion and translocation events. Furthermore, all patients demonstrated unique *AR*-GSR breakpoint locations. Interestingly, patient A27 displayed a rearrangement that deleted half of exon 4 as well as exons 5 and 6 and was the only patient whose *AR*-GSR was also detected from the RNA-seq sample (Supplementary Fig. S7). This rearrangement may lead to translation of a truncated, constitutively active protein product, and could thus have some biological relevance. None of the *AR*-GSRs detected by our pipeline were associated with the expression of previously known *AR-V*s and their variant allele fractions were relatively low (range 2.6–10.9%).

### Expression of AR-V7 is heterogeneous at the protein level

To study how the detected differences in AR variant expression between PC stages are translated to the protein level, we performed immunohistochemistry against AR-V7 with tumour microarrays of hormone-naïve PC from prostatectomies (*n* = 146), locally recurrent CRPCs (*n* = 97) and metastatic CRPC samples (103 metastases in total from 31 patients; 1–5 metastases per patient). We also studied immunohistochemistry of AR (N-terminal antibody recognising full-length AR as well as all variants containing exon 1, including *AR-V3*, *AR-V7* and *AR-V9*). As a positive control we used a sample of 22Rv1 cell line known to contain high AR-V7 expression (Supplementary Fig. S8a). Primarily, AR-V7 was detected in the nucleus (92% of hormone-naïve, 62% of CRPC and 75% of metastatic samples), although variable cytoplasmic staining could be detected in minority of the samples in all phases of the disease (12% of hormone-naïve, 32% of CRPC and 21% of metastatic samples) (Supplementary Fig. S8b). In contrast to AR staining, the AR-V7 staining was heterogeneous and often present in only a fraction of the cells (Supplementary Fig. S8b). For example, 89% of the positive, hormone-naïve cases had nuclear AR-V7 in less than 10% of the cells (mean value of positive cells 6.4%, median 3.2%) (Supplementary Fig. S9a,b). In CRPC, the number of AR-V7-negative cases increased as compared to hormone-naïve disease (38% vs. 8% of no nuclear AR-V7 detected, respectively) (Supplementary Fig. S9a). Interestingly, many of the AR-V7-negative CRPC samples had a strong mesenchymal phenotype, while the cells in most positive tumours had round, epithelial phenotype. In the positive CRPC cases, the percentage of AR-V7-positive cells increased as compared to hormone-naïve disease, with mean value 24.9% and median 13.6% (Supplementary Fig. S9b). As for the metastatic disease, 88% of the tumours studied had detectable AR-V7 positivity, and all 31 patients had one or more AR-V7-positive metastases. It should be noted that direct comparison of AR-V7 mRNA and protein levels is not possible in most of the cases as samples do not originate from the same tumour areas. However, general observations can be made. For example, patient A28 with highest *AR-V7* expression at the mRNA level in the metastasis subjected to sequencing analysis (Fig. 1) had AR-V7 positivity in all four metastases that were studied with immunohistochemistry.

## Discussion

This study describes the *AR* aberration status in two comprehensive patient cohorts including specimens from BPH, untreated localised and metastatic PC as well as both locally recurrent and metastatic CRPCs. We show that even though *AR-V3*, *AR-V7* and *AR-V9* are expressed widely in different sample types, they are statistically more highly expressed in metastatic CRPCs in comparison to two hormone-naïve sample groups, prostatectomies and lymph node metastases. This further reinforces the conception that AR-Vs likely have a role in CRPC progression and development of resistance to AR-targeted therapies.

**Fig. 4:**
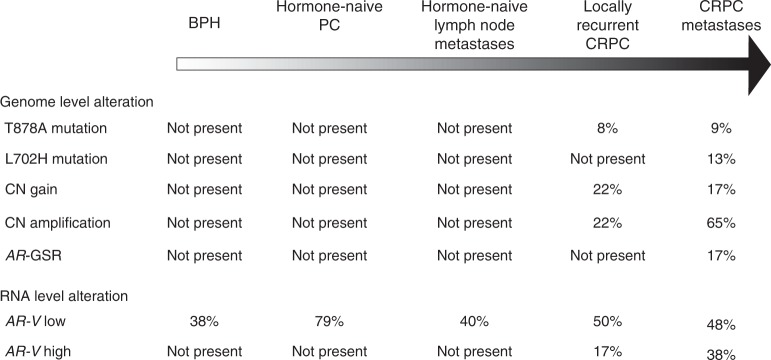
Summary of the frequency of the genome and RNA level alterations of *AR* during different stages of prostate cancer. Copy number (CN) changes of *AR* are presented as gains (>1 copy of *AR*) and amplifications (>2 *AR* copies). *AR-V* expression levels are divided into *AR-V* low (<5% of splice variant of *AR* transcript) and *AR-V* high (>5% of splice variant of *AR* transcript) groups. The data are from MiSeq assays for all other sample groups, except for BPH and locally recurrent CRPC whose data are from HiSeq assays

In CRPC metastases, the expression of *AR-V7* was 13% of *AR* transcript at maximum, and it was present in 21/29 cases, whereas the expression levels of *AR-V3* and *AR-V9* were highly similar (7% of *AR* transcript at maximum) and detected in 23/29 and 22/29 CRPC metastases, respectively. Our finding that *AR-V3*, *AR-V7* and *AR-V9* are present at varying levels also in benign prostate tissue and hormone-naïve primary PCs is in line with previous reports.^23,50^ Furthermore, our whole-genome and targeted DNA sequencing results were in accordance with previous reports demonstrating that *AR* mutations and amplifications are rare in early stages of untreated PC, but occur much more frequently in patients affected by metastatic CRPC.^4,23,50^ In our study, no *AR* mutations or copy number changes were detected in untreated cases; they were observed only in locally recurrent and metastatic CRPC specimens. In CRPC metastases, 5/23 cases harboured an *AR* mutation and 19/23 cases had a copy number gain or amplification. Out of 22 cases of metastatic CRPC of which both DNA- and RNA-seq data were available, all but one patient (patient A5) had at least one *AR* aberration underlining the crucial role of AR in the disease progression. Figure 4 summarises both genome and RNA level alterations of *AR* detected in this study during different stages of PC.

It is noteworthy that lymph node metastasis specimens from patients who had not undergone any ADT did not show elevated levels of *AR-V*s. It has been demonstrated earlier using several PC cell lines that inhibition of the full-length AR protein via castration, antiandrogen treatment or siRNA induced the expression of AR-V7, although concomitant, yet lesser increases in full-length AR were also observed.^51,52^ It has also been shown that ADT does not directly regulate levels of AR-V7, but rather enhances *AR* gene transcription rate and splicing factor recruitment to *AR* pre-mRNA, thus elevating AR-V7 levels.^53^ Accordingly, we also showed that the expression levels of *AR-V7* as well as levels of *AR-V3* and *AR-V9* were strongly associated with the levels of full-length *AR,* indicating that the *AR-V* expression is dependent on transcription rate of *AR* locus.

Interestingly, we observed that *AR-V7* was strongly co-expressed with *AR-V9* in sample set 2, which is in line with a recent report also demonstrating simultaneous expression of *AR-V7* and *AR-V9* in CRPC metastases.^30^ Moreover, our data showed that *AR-V7* was co-expressed with *AR-V3*; and there was also a clear positive correlation between expression of *AR-V9* and *AR-V3*. It should be noted that out of 25 CRPC metastases that expressed any *AR* variant, as many as 17 cases harboured expression of all three of these *AR-V*s. Since *AR-V3*, *AR-V7* and *AR-V9* are constitutively active, it is reasonable to expect that their combined contribution to PC progression might be greater than what could be expected when their effects are studied separately. In our data, *AR-V* expression levels were above 5% when compared to overall *AR* transcript expression levels in 11 metastatic CRPC specimens (sample set 2). Since metastatic CRPC specimens harboured three times higher expression of *AR-FL* in comparison to hormone-naïve prostatectomy samples it would mean that 5% *AR-V* fraction does not yet bring the *AR-V* levels to corresponding levels of *AR-FL* in hormone-naïve PC. However, the levels of *AR-V* required to drive an androgen-independent transcriptome are unknown.

It has been demonstrated in several cell line studies that AR-Vs are able to induce the expression of AR-controlled genes such as *KLK3*, *TMPRSS2* and *FKBP5* in the absence of androgens or AR-FL.^19,20,54,55^ Therefore, we interrogated the levels of classical AR-regulated genes in our sample sets, and calculated the summed *z*-score of five androgen responsive genes (*KLK3*, *FKBP5*, *TMPRSS2*, *ACPP* and *SLC45A3*). There was no association between the expression levels of *AR-V3*, *AR-V7* or *AR-V9* and *z*-score in sample set 2. Likewise, no correlation was detected when *KLK3* expression was compared to the expression levels of these *AR-V*s in CRPC metastases. One explanation for this discrepancy could be the fact that metastatic CRPC samples expressing the highest levels of *AR-V*s were taken at autopsy making it highly unlikely that AR-regulation was anymore classical at the late stage of the disease. In addition, it has previously been demonstrated that bone metastases with high *AR-V* levels did not show high levels of *KLK3*, *KLK2*, *FKBP5*, *TMPRSS2* and *NKX3-1*, whereas the levels of other transcripts known to be positively regulated by AR were elevated (including *CDK1*, *CYCLINA2*, *HSP27* and *C-MYC*).^56^ Therefore, it seems that the expression profile induced by AR-Vs can be context-dependent and might not correspond to the effects observed in cell lines.

As protein expression does not always fully correlate with mRNA expression, and as the AR-Vs may also be regulated post-transcriptionally, it is of importance to study their expression also at the protein level. We assessed the expression of AR-V7 by immunohistochemistry in hormone-naïve PC, locally recurrent CRPC and metastatic CRPC samples. Although a third of CRPCs in this cohort were found negative for AR-V7 protein, the results support the view that AR-V7 expression increases during castration resistance, and that the protein is present in most PC metastases. It is noteworthy that expression of AR-V7 is highly more heterogeneous than that of AR overall. This indicates either differences in transcriptional expression of AR-V7 between tumour cells, or heterogeneous post-transcriptional regulation of it within tumour cell populations.

Genomic structural rearrangements (GSRs) have recently been shown to define a class of *AR* aberrations occurring at a considerable frequency in CRPC material.^31,34^ Henzler et al. studied *AR*-GSRs in 30 rapid autopsy CRPC soft tissue metastases obtained from 15 patients and found that 10/30 metastases (6/15 patients) displayed at least one *AR*-GSR event. Instead, De Laere et al. utilised liquid biopsies from 30 chemotherapy pretreated or chemo-naïve CRPC patients and detected at least one *AR*-GSR in 15/30 patients. We observed *AR*-GSRs in 5/30 patients with metastatic CRPC, which is considerably less when compared to these prior findings. The reason for this discrepancy is unclear, but it can be at least partly due to the fact that there were more uncovered regions in our assay (76–82% of *AR* covered) than in Henzler et al. assay (83–89% of *AR* covered). Interestingly, *AR*-GSRs were not detected in the context of *AR* missense mutations in both our and Henzler et al. sample cohorts.^31^ Furthermore, in our material, the break fusion junctions of *AR*-GSRs were variable, demonstrating several types of rearrangements, but none of the *AR*-GSRs were associated with the expression of previously known *AR-V*s. Patient A27 was the only one whose *AR*-GSR was also detected by RNA-seq and his variant allele fraction was also the highest being 10.9%. For other patients with *AR*-GSRs the variant allele fractions ranged from 2.6 to 8.6%. In CRPC metastases, half of the *AR*-GSR-positive patients expressed *AR-V*s,^31^ whereas all but one of *AR*-GSR-positive patients who were liquid-biopsied harboured *AR-V* expression.^34^ Together, these results demonstrate that the connection of *AR*-GSRs and the expression of *AR-V*s is highly variable in different sample cohorts. It is also noteworthy that in the study of Henzler et al., the only previously reported variant that was associated with the presence of *AR*-GSRs were *AR-V7* and *AR-V12* (*ARv567es*), but the data from De Laere et al. showed that the majority of *AR*-GSR-positive patients expressed multiple previously reported *AR-V*s. *AR*-GSRs were restricted to CRPC specimens in both our and Henzler et al. data, suggesting that they are yet another means of CRPC to retain AR signalling.

In conclusion, the finding that *AR-V* expression levels increase in patients treated with androgen-deprivation therapy might indicate that there is a clonal selection pressure on the different tumour clones in order to maintain functional AR signalling independent of the androgen levels. We provide evidence that *AR-V3*, *AR-V7* and *AR-V9* are co-expressed in metastatic CRPC highlighting the fact that targeting of the AR ligand-binding domain might not be sufficient to achieve clinically relevant treatment responses. Consequently, inhibiting AR function via regions common to all AR-Vs is likely to provide additional benefit to patients with CRPC.

## Electronic supplementary material

Supplementary Table S1

Supplementary Table S2

Supplementary Table S3

Supplementary Table S4

Supplementary Table S5

Supplementary Figure S1

Supplementary Figure S2

Supplementary Figure S3

Supplementary Figure S4

Supplementary Figure S5

Supplementary Figure S6

Supplementary Figure S7

Supplementary Figure S8

Supplementary Figure S9

Supplementary methods

Supplementary files

## Availability of data and material

All data generated or analysed during this study are included in this published article [and its supplementary information files].

## References

[1] Visakorpi T (1995). In vivo amplification of the androgen receptor gene and progression of human prostate cancer. Nat. Genet..

[2] Waltering KK (2009). Increased expression of androgen receptor sensitizes prostate cancer cells to low levels of androgens. Cancer Res..

[3] Brooke GN, Bevan CL (2009). The role of androgen receptor mutations in prostate cancer progression. Curr. Genom..

[4] Grasso CS (2012). The mutational landscape of lethal castration-resistant prostate cancer. Nature.

[5] Cai C, Balk SP (2011). Intratumoral androgen biosynthesis in prostate cancer pathogenesis and response to therapy. Endocr. Relat. Cancer.

[6] Locke JA (2008). Androgen levels increase by intratumoral de novo steroidogenesis during progression of castration-resistant prostate cancer. Cancer Res..

[7] Montgomery RB (2008). Maintenance of intratumoral androgens in metastatic prostate cancer: a mechanism for castration-resistant tumor growth. Cancer Res..

[8] Palmberg C (2000). Androgen receptor gene amplification at primary progression predicts response to combined androgen blockade as second line therapy for advanced prostate cancer. J. Urol..

[9] Gottlieb B, Beitel LK, Nadarajah A, Paliouras M, Trifiro M (2012). The androgen receptor gene mutations database: 2012 update. Hum. Mutat..

[10] Eisermann K, Wang D, Jing Y, Pascal LE, Wang Z (2013). Androgen receptor gene mutation, rearrangement, polymorphism. Transl. Androl. Urol..

[11] Attard G (2009). Selective inhibition of CYP17 with abiraterone acetate is highly active in the treatment of castration-resistant prostate cancer. J. Clin. Oncol..

[12] Tran C (2009). Development of a second-generation antiandrogen for treatment of advanced prostate cancer. Science.

[13] Annala M (2018). Circulating tumor DNA genomics correlate with resistance to abiraterone and enzalutamide in prostate cancer. Cancer Discov..

[14] Romanel A (2015). Plasma AR and abiraterone-resistant prostate cancer. Sci. Transl. Med.

[15] Azad AA (2015). Androgen receptor gene aberrations in circulating cell-free DNA: biomarkers of therapeutic resistance in castration-resistant prostate cancer. Clin. Cancer Res..

[16] Wyatt AW (2016). Genomic alterations in cell-free DNA and enzalutamide resistance in castration-resistant prostate cancer. JAMA Oncol..

[17] Conteduca V (2017). Androgen receptor gene status in plasma DNA associates with worse outcome on enzalutamide or abiraterone for castration-resistant prostate cancer: a multi-institution correlative biomarker study. Ann. Oncol..

[18] Scher HI (2010). Antitumour activity of MDV3100 in castration-resistant prostate cancer: a phase 1-2 study. Lancet.

[19] Dehm SM, Schmidt LJ, Heemers HV, Vessella RL, Tindall DJ (2008). Splicing of a novel androgen receptor exon generates a constitutively active androgen receptor that mediates prostate cancer therapy resistance. Cancer Res..

[20] Hu R (2009). Ligand-independent androgen receptor variants derived from splicing of cryptic exons signify hormone-refractory prostate cancer. Cancer Res..

[21] Lu C, Luo J (2013). Decoding the androgen receptor splice variants. Transl. Androl. Urol..

[22] Jenster G (1991). Domains of the human androgen receptor involved in steroid binding, transcriptional activation, and subcellular localization. Mol. Endocrinol..

[23] Robinson D (2015). Integrative clinical genomics of advanced prostate cancer. Cell.

[24] Antonarakis ES (2015). Androgen receptor splice variant 7 and efficacy of taxane chemotherapy in patients with metastatic castration-resistant prostate cancer. JAMA Oncol..

[25] Antonarakis ES (2014). AR-V7 and resistance to enzalutamide and abiraterone in prostate cancer. N. Engl. J. Med..

[26] Antonarakis ES (2017). Clinical significance of androgen receptor splice variant-7 mRNA detection in circulating tumor cells of men with metastatic castration-resistant prostate cancer treated with first- and second-line abiraterone and enzalutamide. J. Clin. Oncol..

[27] Seitz AK (2017). AR-V7 in peripheral whole blood of patients with castration-resistant prostate cancer: association with treatment-specific outcome under abiraterone and enzalutamide. Eur. Urol..

[28] Steinestel J., et al. Detecting predictive androgen receptor modifications in circulating prostate cancer cells. *Oncotarget* 2015. 10.18632/oncotarget.3925. 10.18632/oncotarget.3925 PMC6609250 31289619

[29] Del Re M, et al. The detection of androgen receptor splice variant 7 in plasma-derived exosomal RNA strongly predicts resistance to hormonal therapy in metastatic prostate cancer patients. *Eur. Urol.* **71**, 680–687 (2017). 10.1016/j.eururo.2016.08.012 27733296

[30] Kohli M., et al. Androgen receptor variant AR-V9 is coexpressed with AR-V7 in prostate cancer metastases and predicts abiraterone resistance. *Clin Cancer Res.* **23**, 4704–4715 (2017). 10.1158/1078-0432.CCR-17-0017 PMC5644285 28473535

[31] Henzler C (2016). Truncation and constitutive activation of the androgen receptor by diverse genomic rearrangements in prostate cancer. Nat. Commun..

[32] Li Y (2011). Intragenic rearrangement and altered RNA splicing of the androgen receptor in a cell-based model of prostate cancer progression. Cancer Res..

[33] Li Y (2012). AR intragenic deletions linked to androgen receptor splice variant expression and activity in models of prostate cancer progression. Oncogene.

[34] De Laere B., et al. Comprehensive profiling of the androgen receptor in liquid biopsies from castration-resistant prostate cancer reveals novel intra-AR structuralvariation and splice variant expression patterns. *Eur. Urol.* **72**, 192–200 (2017). 10.1016/j.eururo.2017.01.011 28104311

[35] Bova GS (1993). Homozygous deletion and frequent allelic loss of chromosome 8p22 loci in human prostate cancer. Cancer Res..

[36] Annala M (2015). Recurrent SKIL-activating rearrangements in ETS-negative prostate cancer. Oncotarget.

[37] Gundem G (2015). The evolutionary history of lethal metastatic prostate cancer. Nature.

[38] Bova GS (2016). Integrated clinical, whole-genome, and transcriptome analysis of multisampled lethal metastatic prostate cancer. Cold Spring Harb. Mol. Case Stud..

[39] Langmead B, Salzberg SL (2012). Fast gapped-read alignment with Bowtie 2. Nat. Methods.

[40] Li H (2009). The sequence alignment/map format and SAMtools. Bioinformatics.

[41] Wang K, Li M, Hakonarson H (2010). ANNOVAR: functional annotation of genetic variants from high-throughput sequencing data. Nucleic Acids Res..

[42] Quinlan AR, Hall IM (2010). BEDTools: a flexible suite of utilities for comparing genomic features. Bioinformatics.

[43] Kim D (2013). TopHat2: accurate alignment of transcriptomes in the presence of insertions, deletions and gene fusions. Genome Biol..

[44] Leinonen KA (2013). Loss of PTEN is associated with aggressive behavior in ERG-positive prostate cancer. Cancer Epidemiol. Biomark. Prev..

[45] Lallous N (2016). Functional analysis of androgen receptor mutations that confer anti-androgen resistance identified in circulating cell-free DNA from prostate cancer patients. Genome Biol..

[46] Zhou J, Liu B, Geng G, Wu JH (2010). Study of the impact of the T877A mutation on ligand-induced helix-12 positioning of the androgen receptor resulted in design and synthesis of novel antiandrogens. Proteins.

[47] Taplin ME (1999). Selection for androgen receptor mutations in prostate cancers treated with androgen antagonist. Cancer Res..

[48] Carreira S (2014). Tumor clone dynamics in lethal prostate cancer. Sci. Transl. Med.

[49] Zhao XY (2000). Glucocorticoids can promote androgen-independent growth of prostate cancer cells through a mutated androgen receptor. Nat. Med..

[50] Cancer Genome Atlas Research Network. The molecular taxonomy of primary prostate. *Cancer Cell.* **163**, 1011–1025 (2015). 10.1016/j.cell.2015.10.025 PMC4695400 26544944

[51] Yu Z (2014). Rapid induction of androgen receptor splice variants by androgen deprivation in prostate cancer. Clin. Cancer Res..

[52] Hu R (2012). Distinct transcriptional programs mediated by the ligand-dependent full-length androgen receptor and its splice variants in castration-resistant prostate cancer. Cancer Res..

[53] Liu LL (2014). Mechanisms of the androgen receptor splicing in prostate cancer cells. Oncogene.

[54] Guo Z (2009). A novel androgen receptor splice variant is up-regulated during prostate cancer progression and promotes androgen depletion-resistant growth. Cancer Res..

[55] Sun S (2010). Castration resistance in human prostate cancer is conferred by a frequently occurring androgen receptor splice variant. J. Clin. Invest..

[56] Hornberg E (2011). Expression of androgen receptor splice variants in prostate cancer bone metastases is associated with castration-resistance and short survival. PLoS One.

