# Supplementary material for: Constitutively active androgen receptor splice variants AR-V3, AR-V7 and AR-V9 are co-expressed in castration-resistant prostate cancer metastases
Source: Br J Cancer. 2018 Jul 10;119(3):347–56. doi: 10.1038/s41416-018-0172-0 (PMC6070921; doi:10.1038/s41416-018-0172-0)
Supplement: Supplementary file 2 — Supplementary Table S2 [file 41416_2018_172_MOESM2_ESM.docx]

Supplementary Table S2. Unique splice junctions for *AR-V* detection.

| *AR-V* | Detected splice junction |
| --- | --- |
| *AR-V3* | exon 2 – CE4 |
| *AR-V4* | exon 3 – CE4 |
| *AR-V5* | exon 3 – CE2-1* |
| *AR-V6* | exon 3 – CE2-2* |
| *AR-V7* | exon 3 – CE3 |
| *AR-V9* | exon 3 – CE5 |
| *AR-V12* | exon 4 – exon 8 |

*Alternative 5’ splicing site
