# Supplementary material for: Constitutively active androgen receptor splice variants AR-V3, AR-V7 and AR-V9 are co-expressed in castration-resistant prostate cancer metastases
Source: Br J Cancer. 2018 Jul 10;119(3):347–56. doi: 10.1038/s41416-018-0172-0 (PMC6070921; doi:10.1038/s41416-018-0172-0)
Supplement: Supplementary file 5 — Supplementary Table S5 [file 41416_2018_172_MOESM5_ESM.docx]

| **Patient**  **ID** | ***AR* copy**  **number** | **Tumour type** | **Tissue** | ***AR*-GSR** | **Break fusion junction coordinates (hg19)** | **Remarks** | **# supporting split reads** | **Variant allele fraction** |
| --- | --- | --- | --- | --- | --- | --- | --- | --- |
| A4 | 68 | CRPC metastasis | Liver | Inversion | chrX:66,189,781 /  chrX:66,863,033 | 5’ breakpoint located in a *LINE-1* element between *EDA2R* and *AR*, region containing exon 1, 1b and most of intron 1 inverted | 22 | 8.6% |
| A7 | 6 | CRPC metastasis | Subdural | Inversion | chrX:66,783,645 /  chrX:66,784,142 | Inverted ~500bp region in *AR* intron 1 between exon 1 and 1b | 53 | 5.4% |
| A8 | 59 | CRPC metastasis | Liver | Translocation | chr7:35,711,637 /  chrX:66,911,250 | 5’ breakpoint located in a *LINE-1* element in *HERPUD2* intron 3, 3’ breakpoint in *AR* intron 3 -> deletion of cryptic exons CE3/CE5 + exons 4-8 | 18 | 2.6% |
| A11 | 7 | CRPC metastasis | Inguinal lymph node | Duplication | chrX:116,819,638 / chrX:66,769,235 | *AR* intron 1 breakpoint between exons 1 and 1b (exon 1 deleted) | 32 | 6.2% |
| A27 | 9 | CRPC metastasis | Axillary lymph node | Deletion | chrX:66,931,389 / chrX:66,942,180 | Deletes half of exon 4 + exons 5 and 6; detected also from RNA-seq sample | 37 | 10.9% |

Supplementary Table 5. *AR* genomic structural rearrangements detected in metastatic CRPC specimens.
