# Supplementary figures and images for: Constitutively active androgen receptor splice variants AR-V3, AR-V7 and AR-V9 are co-expressed in castration-resistant prostate cancer metastases
Source: Br J Cancer. 2018 Jul 10;119(3):347–56. doi: 10.1038/s41416-018-0172-0 (PMC6070921; doi:10.1038/s41416-018-0172-0)

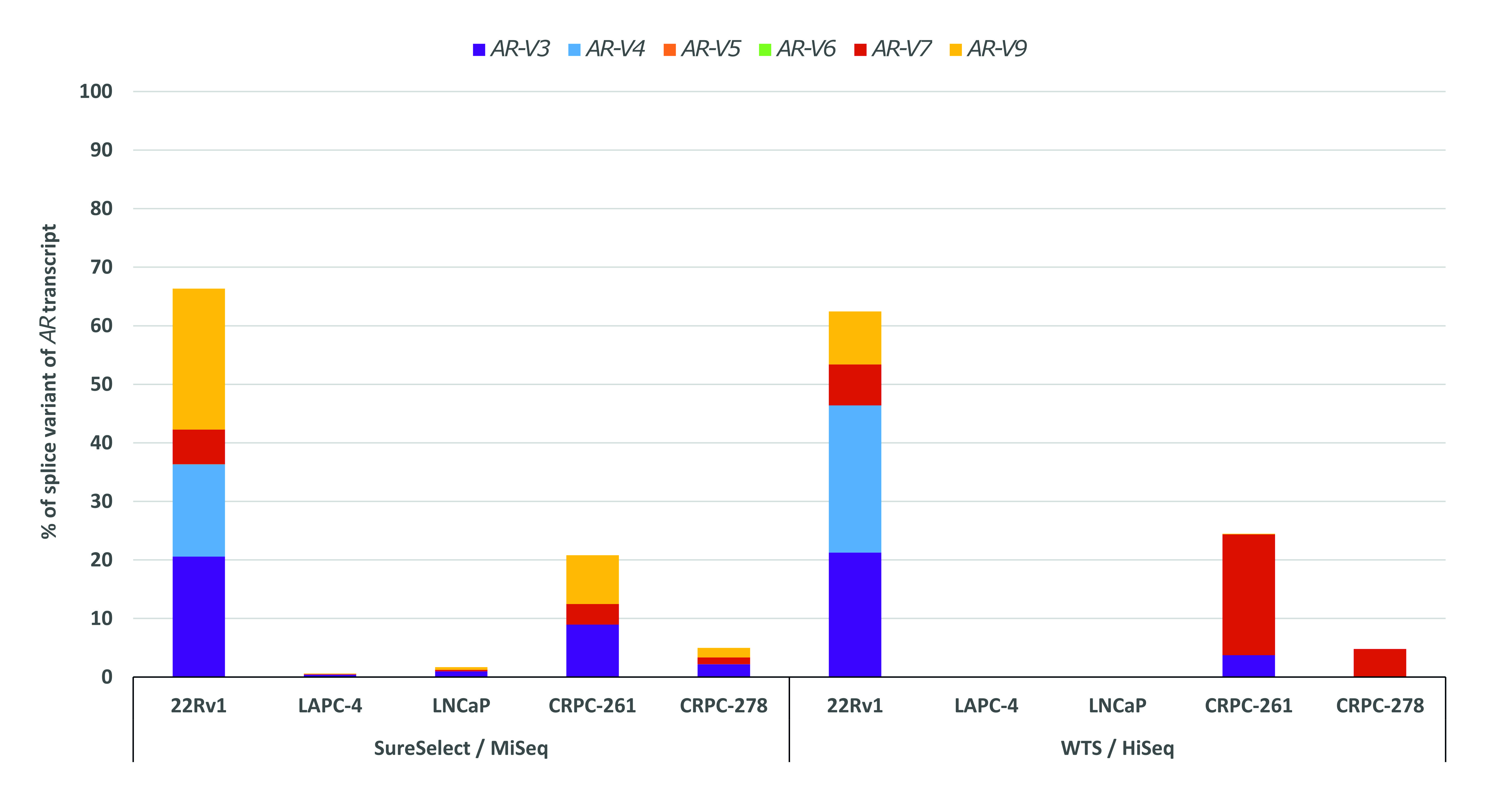

Supplement: Supplementary file 6 — Supplementary Figure S1 [file 41416_2018_172_MOESM6_ESM.jpg]

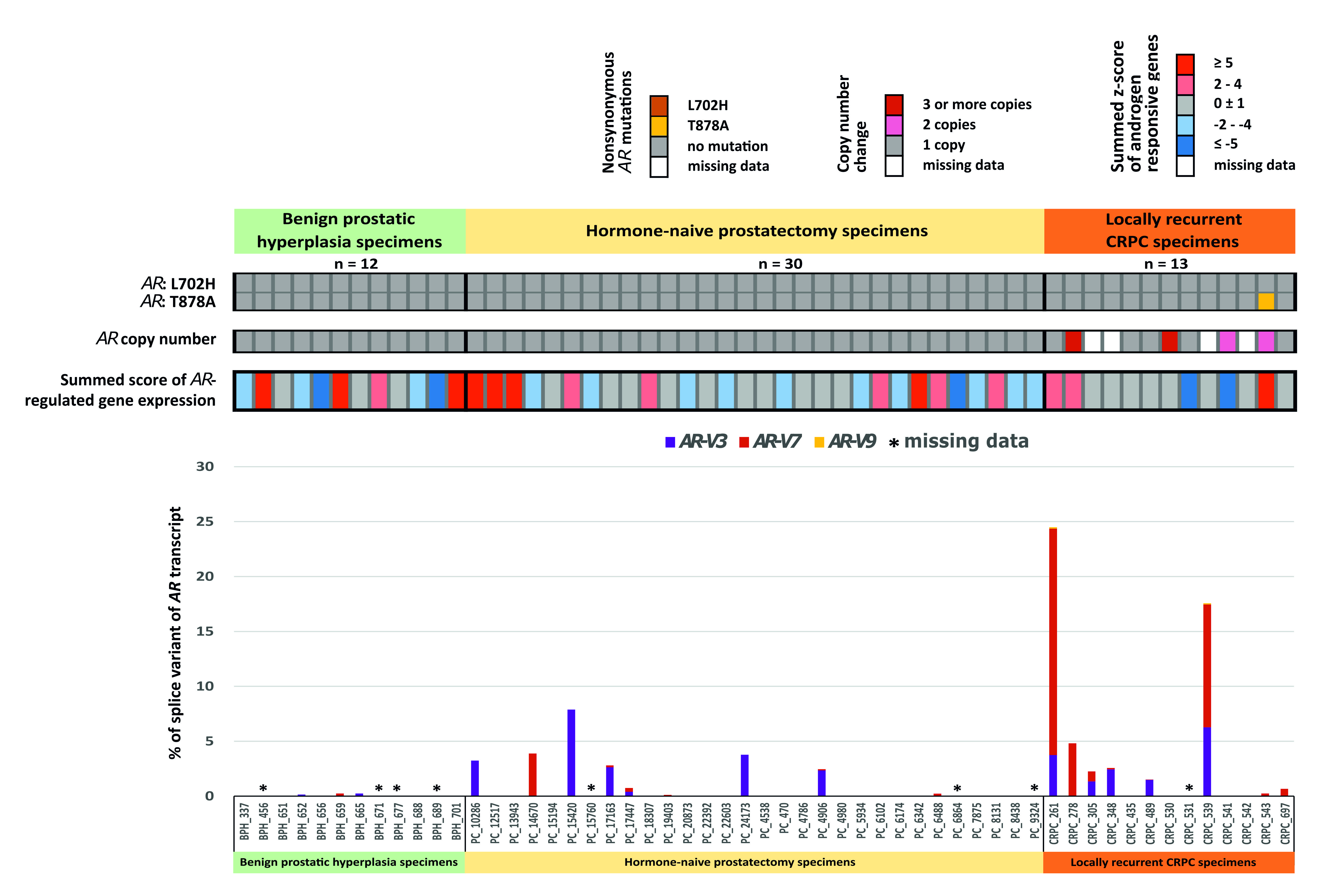

Supplement: Supplementary file 7 — Supplementary Figure S2 [file 41416_2018_172_MOESM7_ESM.jpg]

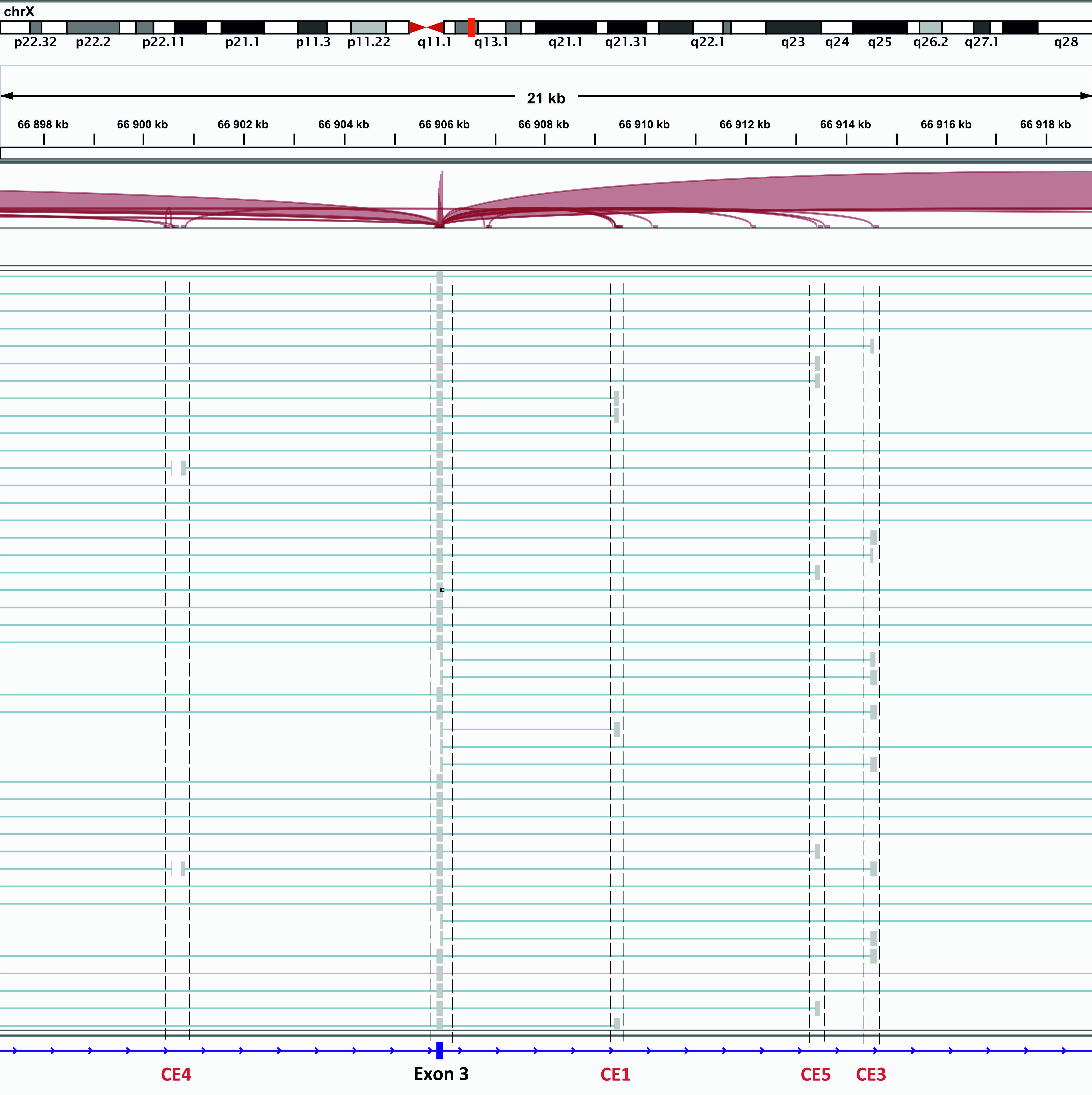

Supplement: Supplementary file 8 — Supplementary Figure S3 [file 41416_2018_172_MOESM8_ESM.jpg]

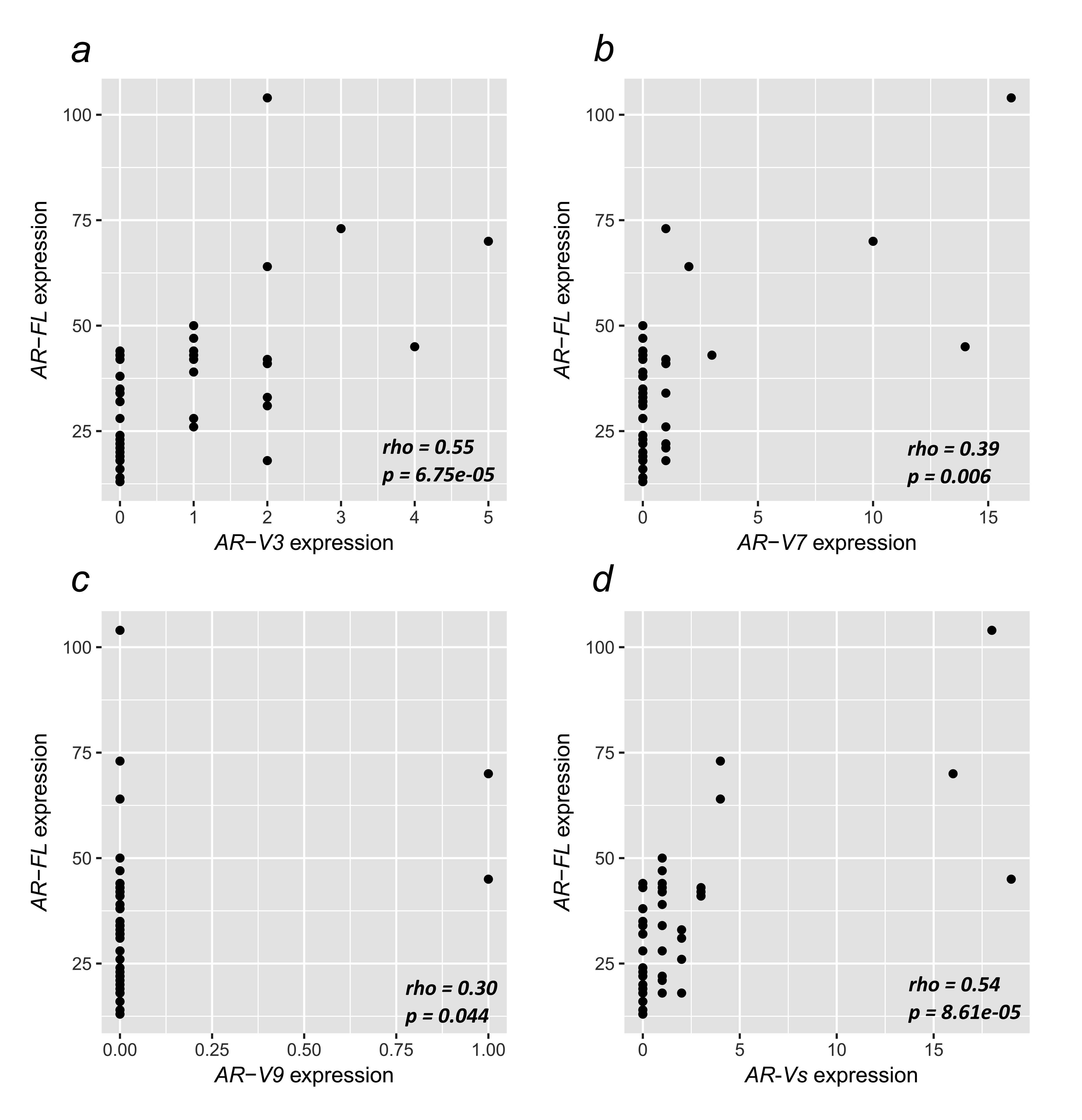

Supplement: Supplementary file 9 — Supplementary Figure S4 [file 41416_2018_172_MOESM9_ESM.jpg]

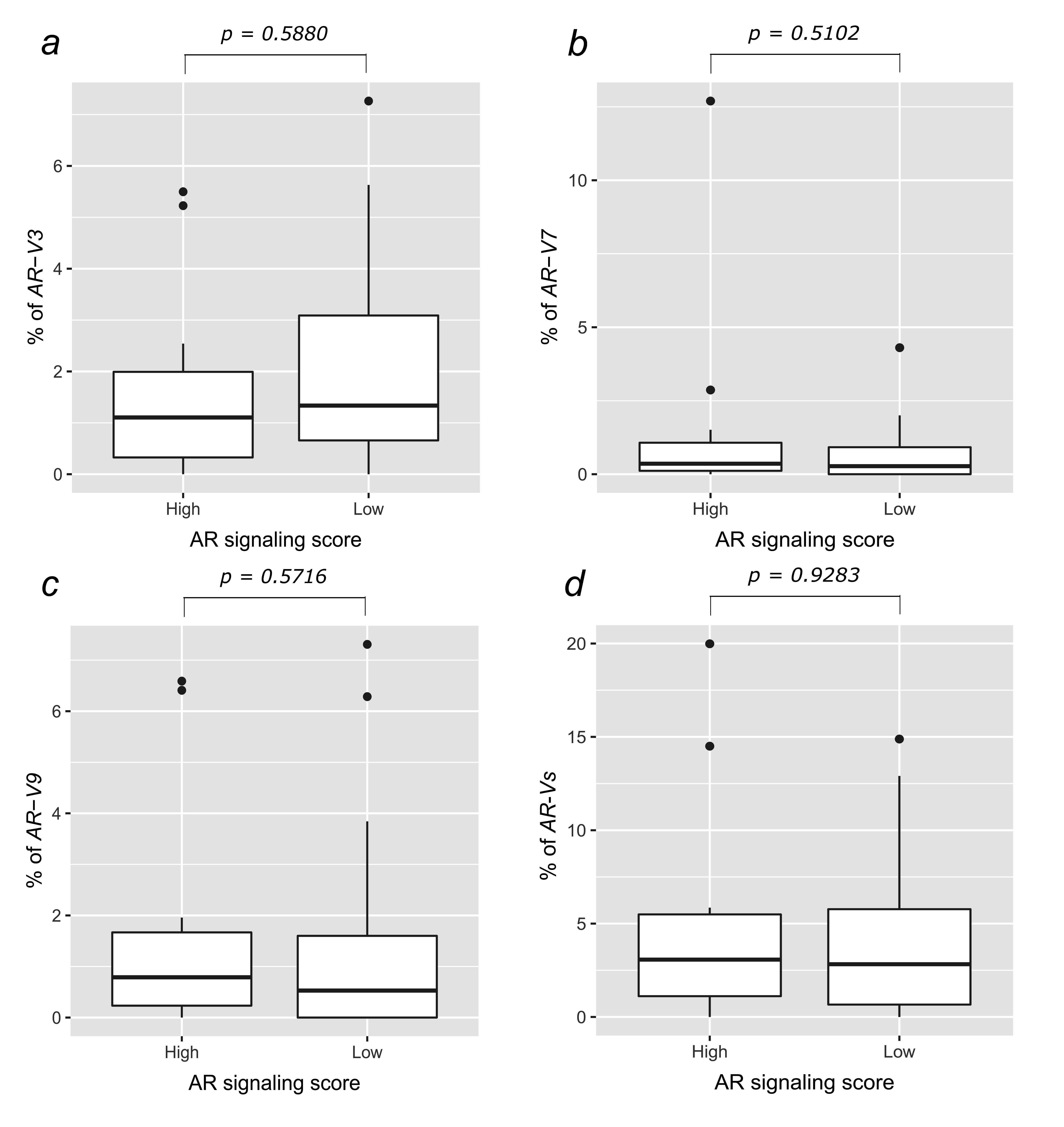

Supplement: Supplementary file 10 — Supplementary Figure S5 [file 41416_2018_172_MOESM10_ESM.jpg]

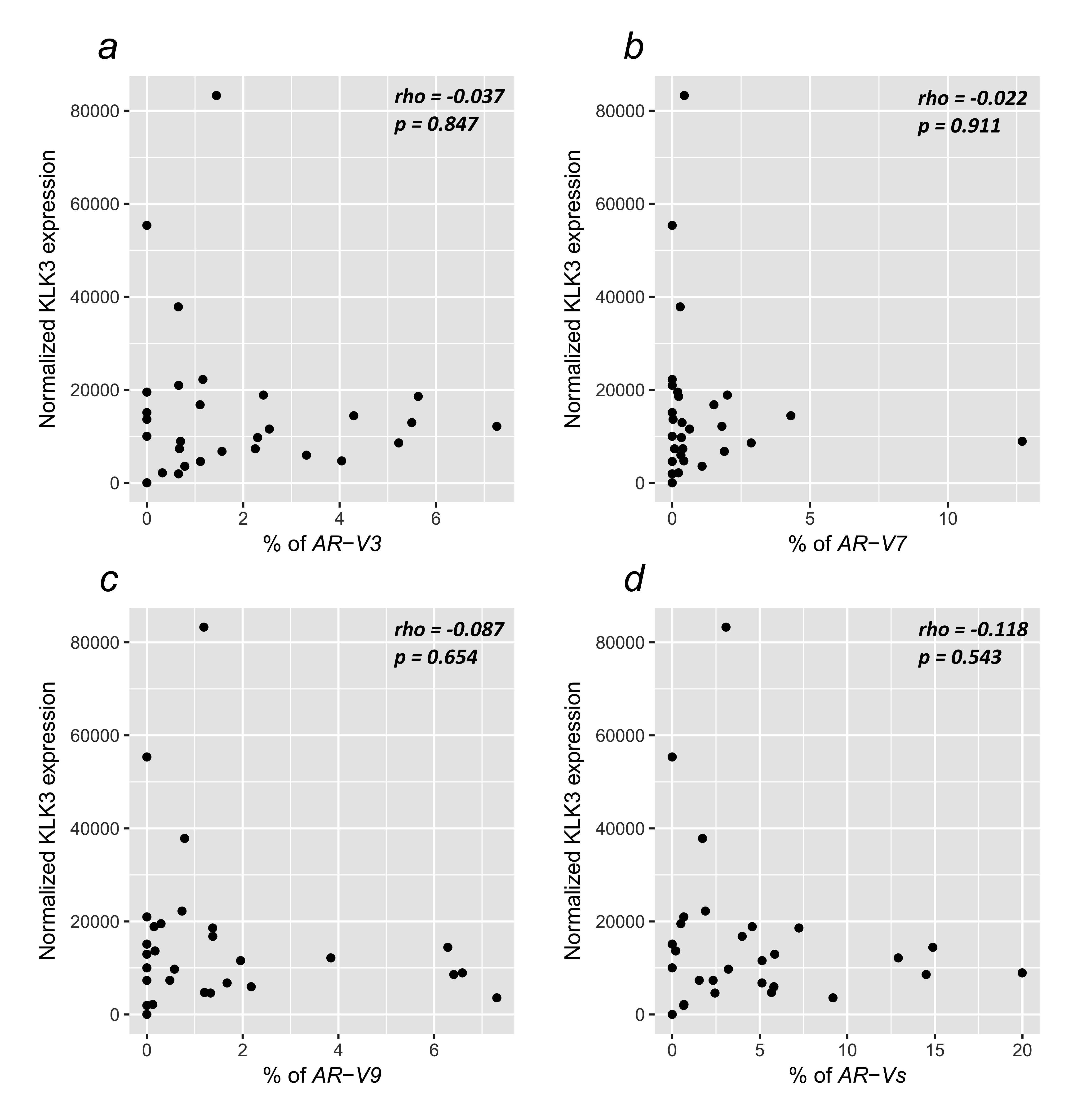

Supplement: Supplementary file 11 — Supplementary Figure S6 [file 41416_2018_172_MOESM11_ESM.jpg]

*a*

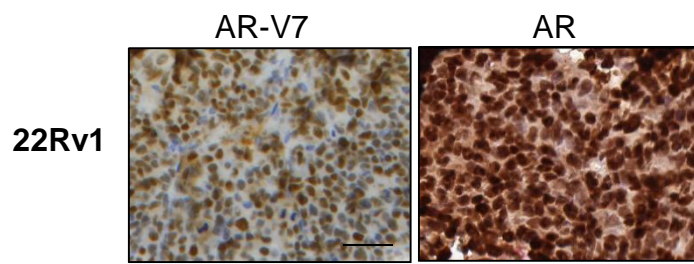

*b*

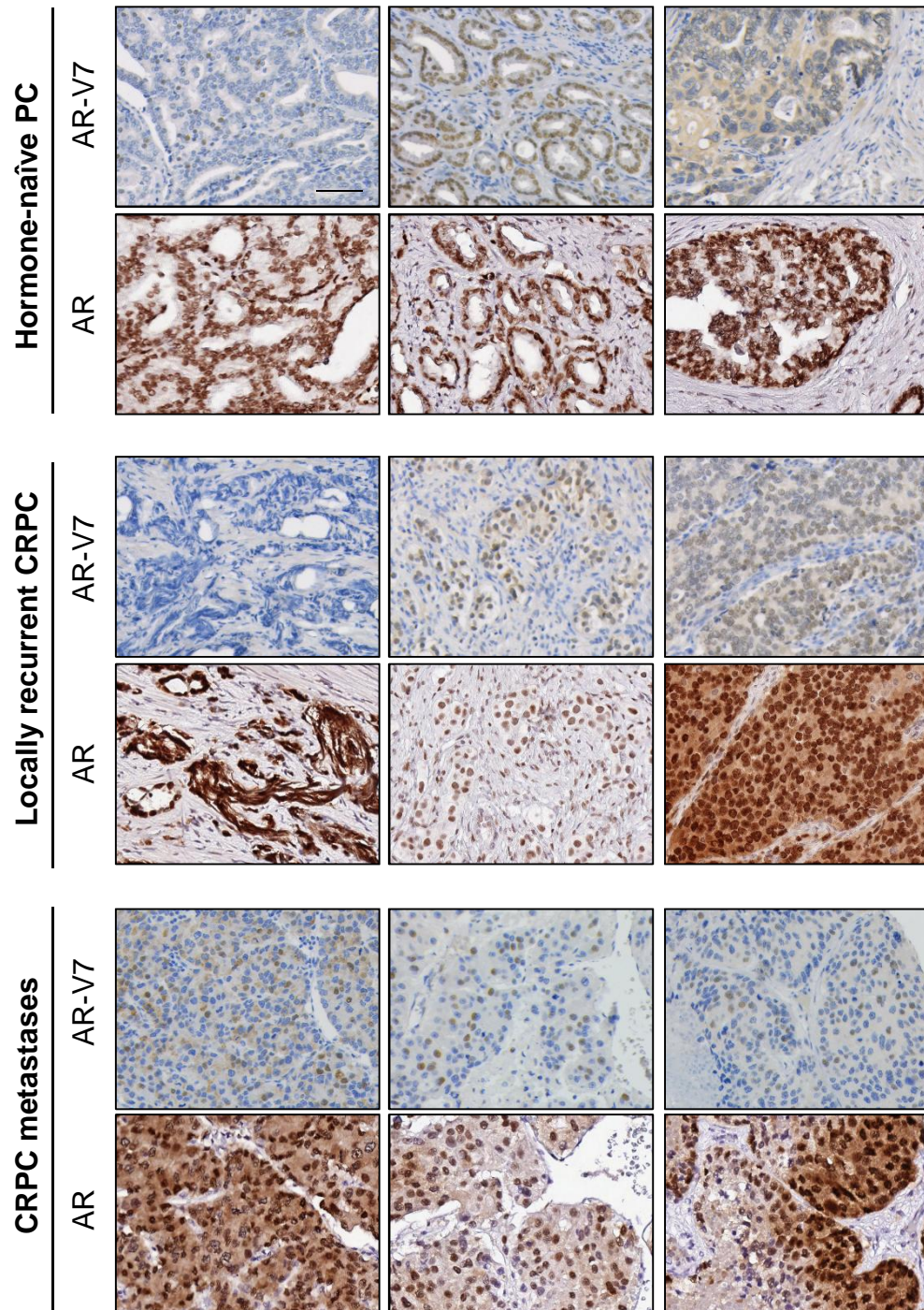

Supplement: Supplementary file 13 — Supplementary Figure S8 [file 41416_2018_172_MOESM13_ESM.pdf]

*a*

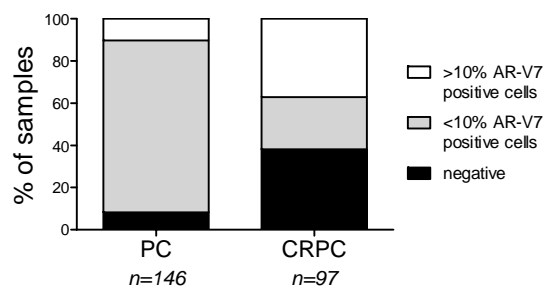

*b*

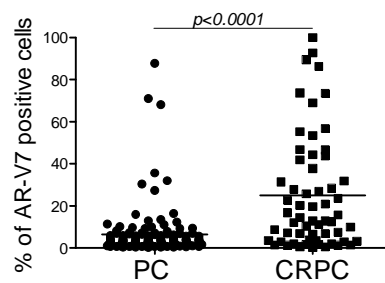

Supplement: Supplementary file 14 — Supplementary Figure S9 [file 41416_2018_172_MOESM14_ESM.pdf]
