# Supplementary material for: Constitutively active androgen receptor splice variants AR-V3, AR-V7 and AR-V9 are co-expressed in castration-resistant prostate cancer metastases
Source: Br J Cancer. 2018 Jul 10;119(3):347–56. doi: 10.1038/s41416-018-0172-0 (PMC6070921; doi:10.1038/s41416-018-0172-0)
Supplement: Supplementary file 15 — Supplementary methods [file 41416_2018_172_MOESM15_ESM.docx]

**Supplementary methods: Bioinformatics**

For analysis of targeted DNA-seq data, Illumina MiSeq reads were aligned to GRCh37 (hg19) genome using Bowtie2 (version 2.2.4)^1^. PCR duplicates were removed using samblaster (version 0.1.22)^2^. Sequencing coverage of the target regions was computed using bedtools (version 2.26.0)^3^. *AR*, *FOXA1* and *SPOP* variants were called using an in-house pipeline that utilizes samtools mpileup^4^, ignoring low-quality alignments (MAPQ > 10). As in some samples *AR* may be highly amplified with mutation present in only one or few of the gene copies, low alternate allele fraction (0.01) and at least three reads showing an alternate allele were required for calling a variant in order to prevent filtering out those variants. For samples without matched normal tissue sample, the variants found in over half of the samples as well as variants annotated in the 1000 Genomes Project were discarded to exclude common polymorphisms. In addition, Pearson’s χ2 test was used to assess the probability that all samples displayed the same underlying allele fraction, which would indicate a sequencing error. For samples with matched normal tissue sample, these were used in the analysis for determining the true, cancer-related mutations, with variants found in the 1000 Genomes project also discarded. The ANNOVAR software^5^ was used for annotation of the called variants. Variants in dataset 1 were analyzed from the whole transcriptome sequencing data similarly.

*AR* copy numbers were analyzed by calculating aligned read counts within overlapping 400 bp windows along the targeted regions using bedtools. Sample-specific differences in overall coverage were corrected by using the median of ratios of the coverage in copy-neutral chromosomal regions (chromosomes 4, 11, 15 and 19) as the normalization factors. The corrected baits coverage values were further normalized using median of ratios normalization method to correct for bait-specific differences in capture efficiency. The bait coverage ratios were obtained by dividing each normalized bait coverage value by the median of all normalized bait coverage values. The estimates of *AR* copy numbers were obtained by taking the median of all *AR* bait coverage ratios. The cutoff for amplification was >1.5 and for deletion <0.5.

Chromosomal rearrangements were called using the in-house Breakfast algorithm. Briefly, the software looks for paired-end reads and individual mates overlapping a chromosomal breakpoint, identifying rearrangements at sites with multiple reads of evidence. 30 bp anchor sequences flanking the breakpoint junction were required for the detection, and minimum evidence required was ten individual reads spanning the junction.

For *AR* splice variant analysis using targeted RNA-seq data, an indexed reference fasta file was constructed containing unique signature sequences for various *AR-V*s as well as *AR-FL*. The signatures consisted of 130 bp of the 3’ end of upstream exon and 130 bp of the 5’ end of downstream exon of a given unique splice junction. In the case of exon 3 as the upstream exon, sequence from the 3’ end of exon 2 was also included as the length of exon 3 is shorter than 130 bp. Illumina MiSeq reads were aligned to this reference using Bowtie2 (version 2.2.4). For a read to be considered as evidence for a splice variant expression, it was required to overlap each of the exons at least 20 bp. Relative *AR-V* expression was estimated as the percentage of all *AR* transcripts by dividing the number of reads aligned to a given *AR-V* signature by the total number of reads aligning to all the splice junctions containing the same upstream exon (estimate of all *AR* transcript expression). For the *AR* splice variant analysis using whole transcriptome RNA-seq data, the HiSeq reads were aligned as above. Since the HiSeq reads were considerably shorter than the MiSeq reads (90 bp vs 150 bp), a shorter 12 bp overlap on each splice junction exon was required for a valid evidence. As the sequencing depth at splice junctions varied substantially between the samples, binomial 95% confidence intervals for the splice variant fractions were estimated using R binom package, taking into account the total number of mapped reads at a given splice junction. The lower bounds of these confidence intervals were then used for sample comparisons instead of the fractions calculated from raw reads; thus, the analysis results that are shown indicate the percentages of splice variants that a given sample at least contains with 95% confidence. The obtained splice variant percentages were further corrected for the tumor cell proportion by taking into account the baseline splice variant expression observed in normal prostate cells.

Expression levels of known AR-regulated genes *FKBP5*, *SGK1*, *KLK3*, *TMPRSS2*, *ACPP* and *LC45A3* that were targeted in the RNA-sequencing panel were quantified and used for determining the AR signaling score. For that, RNA-seq reads were aligned to GRCh37 genome using TopHat2 (version 2.0.13)^6^. Raw read counts were normalized to the expression of three house-keeping genes, *TBP*, *DDX1* and *STARD7*, using median of ratios normalization. For each sample, a Z-score was computed for the expression of each of the genes by subtracting the pooled mean from the RNA-seq expression values and dividing by the pooled standard deviation. The AR signaling score was then computed as the sum of the Z-scores of all six AR-regulated genes.

References

1. Langmead B, Salzberg SL. Fast gapped-read alignment with Bowtie 2. *Nat Methods* 2012; **9**: 357-359.

2. Faust GG, Hall IM. SAMBLASTER: fast duplicate marking and structural variant read extraction. *Bioinformatics* 2014; **30**: 2503-2505.

3. Quinlan AR, Hall IM. BEDTools: a flexible suite of utilities for comparing genomic features. *Bioinformatics* 2010; **26**: 841-842.

4. Li H, Handsaker B, Wysoker A, Fennell T, Ruan J, Homer N *et al.* The Sequence Alignment/Map format and SAMtools. *Bioinformatics* 2009; **25**: 2078-2079.

5. Wang K, Li M, Hakonarson H. ANNOVAR: functional annotation of genetic variants from high-throughput sequencing data. *Nucleic Acids Res* 2010; **38**: e164.

6. Kim D, Pertea G, Trapnell C, Pimentel H, Kelley R, Salzberg SL. TopHat2: accurate alignment of transcriptomes in the presence of insertions, deletions and gene fusions. *Genome Biol* 2013; **14**: R36.
