# Supplementary material for: Constitutively active androgen receptor splice variants AR-V3, AR-V7 and AR-V9 are co-expressed in castration-resistant prostate cancer metastases
Source: Br J Cancer. 2018 Jul 10;119(3):347–56. doi: 10.1038/s41416-018-0172-0 (PMC6070921; doi:10.1038/s41416-018-0172-0)
Supplement: Supplementary file 16 — Supplementary files [file 41416_2018_172_MOESM16_ESM.docx]

**Supplementary files**

Supplementary Table S1. Clinicopathological characteristics of the prostate cancer cases and treatments of CRPC cases from sample set 1 and 2. Detailed treatment modalities are shown for metastatic CRPC patients that harbored *AR* mutations.

Supplementary Table S2. Unique splice junctions for *AR-V* detection.

Supplementary Table S3. Statistical comparison of individual *AR* variants in different sample types from sample set 2 using two-tailed, unpaired Mann-Whitney U test.

Supplementary Table S4. *AR*, *FOXA1* and *SPOP* mutations detected in sample set 2.

Supplementary Table S5. *AR* rearrangements detected in metastatic CRPC patients.

Supplementary Figure S1. Validation of targeted SureSelect *AR* splicing variant detection assay using cell lines and two CRPC samples. SureSelect assay performance was compared to whole transcriptome sequencing data from this study. *AR-V* expression level as a fraction of *AR* transcript is shown.

Supplementary Figure S2. Combined DNA and RNA sequencing data from sample set 1 assayed by whole genome and whole transcriptome sequencing. *AR* mutations, copy number alterations, summed score of AR-regulated gene expression and *AR-V* expression level as a fraction of *AR* transcript are shown. *AR-V* fractions are shown as CI95 lower bound values.

Supplementary Figure S3. RNA-seq read alignment visualization example of patient A17, showing split reads aligning to exon 3 and one of the cryptic exons CE1, CE3, CE4 and CE5. This type of reads were used for the relative quantification of the *AR-V*s by aligning them to the *AR-V* signature sequence reference.

Supplementary Figure S4. The correlation between *AR-FL* mRNA expression and mRNA expression of (a) *AR-V3*, (b) *AR-V7*, (c) *AR-V9*, (d) all three *AR-V*s combined utilizing specimens from sample set 1. Spearman’s rank correlation coefficients and p-values computed via the asymptotic t approximation are also shown in the figures.

Supplementary Figure S5. The association between AR signaling score and the fraction of (a) *AR-V3*, (b) *AR-V7*, (c) *AR-V9*, (d) all three *AR-V*s combined utilizing specimens from sample set 2. “High” group: summed Z-score is above the mean of all samples. “Low” group: summed Z-score is below the mean of all samples.

Supplementary Figure S6. The correlation between normalised *KLK3* expression and fraction of (a) *AR-V3*, (b) *AR-V7*, (c) *AR-V9*, (d) all three *AR-V*s combined utilizing specimens from sample set 2. Spearman’s rank correlation coefficients and p-values computed via the asymptotic t approximation are also shown in the figures.

Supplementary Figure S7. *AR* genomic structural rearrangement (*AR*-GSR) detected in metastatic CRPC sample from patient A27. The rearrangement resulted in a truncated variant that harbored exons 1-3 and half of exon 4.

Supplementary Figure S8. Example microscopic images of AR and AR-V7 immunohistochemistry. (a) 22Rv1 cells known to contain high AR-V7 were used as a positive control for the stainings of AR-V7 (left panel) and AR (right panel). (b) Three representative tumours with different staining patterns are shown for samples of hormone-naïve PC, locally recurrent CRPC and CRPC metastases with AR-V7 (upper panels) and AR (lower panels). Scale bars 50 μm (a) and 100 μm (b).

Supplementary Figure S9. Results of AR-V7 immunohistochemistry in hormone-naïve PC and locally recurrent CRPC. (a) Percentage of cases with no AR-V7 positive cells (negative) and cases with AR-V7 positive tumour cells below or above 10% of all tumour cells. (b) Percentage of AR-V7 positive tumour cells within the positive samples in (a).

Supplementary methods. Description of full bioinformatics methods.
